# Cloning, characterization, and evolutionary patterns of *KCNQ4* genes in anurans

**DOI:** 10.1002/ece3.11311

**Published:** 2024-04-23

**Authors:** Yang Guo, Yanjun Zhu, Shiyuan Shen, Ningning Lu, Jie Zhang, Xiaohong Chen, Zhuo Chen

**Affiliations:** ^1^ The Observation and Research Field Station of Taihang Mountain Forest Ecosystems of Henan Province, College of Life Sciences Henan Normal University Xinxiang China; ^2^ College of Fisheries Henan Normal University Xinxiang China

**Keywords:** adaptive evolution, amphibians, evolutionary patterns, KCNQ4, purifying selection

## Abstract

Acoustic communication plays important roles in the survival and reproduction of anurans. The perception and discrimination of conspecific sound signals of anurans were always affected by masking background noise. Previous studies suggested that some frogs evolved the high‐frequency hearing to minimize the low‐frequency noise. However, the molecular mechanisms underlying the high‐frequency hearing in anurans have not been well explored. Here, we cloned and obtained the coding regions of a high‐frequency hearing‐related gene (*KCNQ4*) from 11 representative anuran species and compared them with orthologous sequences from other four anurans. The sequence characteristics and evolutionary analyses suggested the highly conservation of the *KCNQ4* gene in anurans, which supported their functional importance. Branch‐specific analysis showed that *KCNQ4* genes were under different evolutionary forces in anurans and most anuran lineages showed a generally strong purifying selection. Intriguingly, one significantly positively selected site was identified in the anuran *KCNQ4* gene based on FEL model. Positive selection was also found along the common ancestor of Ranidae and Rhacophoridae as well as the ancestral *O. tianmuii* based on the branch‐site analysis, and the positively selected sites identified were involved in or near the N‐terminal ion transport and the potassium ion channel functional domain of the KCNQ4 genes. The present study revealed valuable information regarding the KCNQ4 genes in anurans and provided some new insights for the underpinnings of the high‐frequency hearing in frogs.

## Graphical abstract

In this study, we cloned and obtained the coding regions of a high‐frequency hearing‐related gene (KCNQ4) from 11 representative anuran species and determined the evolutionary patterns of KCNQ4 in anurans. The highly conservation and functional importance of the KCNQ4 genes in anurans were determined and the KCNQ4 genes were under different evolutionary forces in different anuran lineages. Evidence of positive selection was detected along some anuran lineages and a few positively selected sites identified were involved in the important functional domains of KCNQ4. Our results here provided valuable information regarding the KCNQ4 genes in anurans and insights for understanding the genetic changes underlying the high‐frequency hearing in frogs.

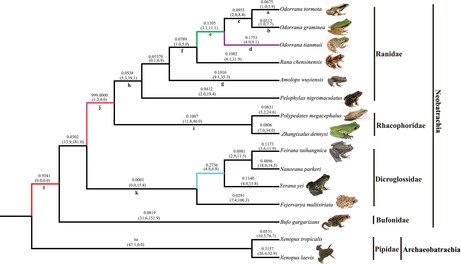

## 1 INTRODUCTION

Anurans (frogs and toads) are one of the most diverse groups of vertebrates (Fei et al., 2009; Frost, 2019). Their transition from water to land and subsequent adaptations (e.g., emergence of lungs, four limbs, and eardrum) to a wide variety of habitats, make anurans evolutionarily significant and remarkable (Fei et al., 2009). Most notably, their hearing system underwent many important morphological and functional adaptations different from fish (Webster et al., 1992). Acoustic communication plays significant roles in the survival, reproduction and evolution of anurans (Chen & Wiens, 2020). However, the perception and discrimination of conspecific sound signals were always affected by masking ambient background noise (Vélez et al., 2013). Interestingly, three torrent frogs, i.e., *Odorrana tormota*, *O. graminea* and *Huia cavitympanum*, were shown to produce and detect ultrasounds to minimize the intense, predominantly low‐frequency masking background noise (Arch & Narins, 2008; Feng et al., 2006; Shen et al., 2011). However, the molecular mechanism underlying the ultrasonic hearing adaptations in anurans is poorly documented to date.

The *KCNQ4* (the voltage‐gated potassium channel subfamily KQT member 4) gene is significantly expressed in the inner ear and neurons of many nuclei of the central auditory pathway, and it encodes a potassium channel protein of approximately 77 kDa (Kharkovets et al., 2000; Kubisch et al., 1999). *KCNQ4* is a candidate gene that regulates electrical signaling and underlies the K^+^ conductance (*I*_*K,n*_) and *g*_*k,L*_ currents at resting potential of the cells, which is associated with the maturation of auditory function (Kharkovets et al., 2000). Previous studies have suggested that the *KCNQ4* gene was associated with high‐frequency hearing, and mutations of *KCNQ4* gene in humans and mice could cause non‐syndromic DFNA2 hereditary deafness (Kharkovets et al., 2006; Kubisch et al., 1999). In addition, previous molecular studies on bat echolocation revealed that the *KCNQ4* gene underwent parallel evolution in echolocating bats (Liu et al., 2011, 2012). Liu et al. (2012) also showed evidence that positive selection occurred on *KCNQ4* in the common ancestors of mammals, suggesting that this gene might have been important for the evolution of mammalian hearing.

Given the important role of *KCNQ4* in high‐frequency hearing, the current study successfully determined the full open reading frame (ORF) sequences of this gene in 11 representative anuran species and compared them with orthologous sequences from other four anurans. The goal of the present study was to test whether evolutionary changes in *KCNQ4* were associated with high‐frequency hearing in anurans. This work sheds insights on the molecular basis underlying the high‐frequency hearing in anurans.

## 2 MATERIALS AND METHODS

### 2.1 Taxonomic coverage

Eleven anuran species were sequenced during this study and detailed taxonomic information is present in Table 1. These samples represent five different microhabitat types (e.g., torrential, aquatic, semi aquatic, arboreal, terrestrial) (Table 1) (Fei et al., 2009). All the samples used in the present study were collected in the wild. These frogs were euthanized by tricaine methanesulfonate (MS‐222) and sacrificed to collect brain tissues. All brain tissues were frozen in liquid nitrogen for 3 h and then stored at −80°C for further use. The animal sampling and use protocols of this study were conducted in accordance with all the ethical guidelines and legal requirements in China, and were approved by the Institutional Care and Ethics Committee of Henan Normal University. The study complies with the ARRIVE guidelines for reporting in Vivo Experiments. In addition, the full‐length ORFs (Open Reading Frames) of *KCNQ4* from *O. graminea* (Lu et al., 2019) and three other anuran species (i.e., *Nanorana parkeri*, *Xenopus laevis* and *X. tropicalis*) were searched and downloaded from the GenBank (http://www.ncbi.nlm.nih.gov) (Table 1). Only the full length form from a species was used in subsequent analyses when multiple splice variants of *KCNQ4* were available.

**TABLE 1:** List of taxonomic samples and *KCNQ4* sequences used in this study.

Family	Scientific name	Accession number	Microhabitat
Ranidae	*Odorrana tormota*	PP179178	Torrential
	*Odorrana graminea*	MK956830	Torrential
	*Odorrana tianmuii*	PP179179	Torrential
	*Rana chensinensis*	PP179180	Terrestrial
	*Amolops wuyiensis*	PP179181	Torrential
	*Pelophylax nigromaculatus*	PP179182	Semi aquatic
Rhacophoridae	*Polypedates megacephalus*	PP179183	Arboreal
	*Zhangixalus dennysi*	PP179184	Arboreal
Dicroglossidae	*Feirana taihangnica*	PP179185	Torrential
	*Yerana yei*	PP179186	Torrential
	*Nanorana parkeri*	XM_018576753.1	Semi aquatic
	*Fejervarya multistriata*	PP179187	Terrestrial
Bufonidae	*Bufo gargarizans*	PP179188	Terrestrial
Pipidae	*Xenopus laevis*	XM_018249576.2	Aquatic
	*Xenopus tropicalis*	XM_012957340.2	Aquatic

### 2.2 Amplification and sequencing of anuran KCNQ4 genes

To clone the coding regions of the KCNQ4 gene from the 11 anuran species mentioned above (Table 1), total RNA was isolated from their brain tissues using RNAiso Plus (TaKaRa, Japan) according to the manufacturer's instructions. RNA integrity and concentrations were determined by electrophoresis on 1% agarose gel and NanoDrop 2000 spectrophotometer (Thermo Scientific, USA), respectively. First‐strand cDNAs were synthesized using the PrimeScript™ II 1st Strand cDNA Synthesis Kit (TaKaRa, Japan) and then stored at −20°C until used. We first amplified partial sequences of the conserved region using the primers based on an alignment of *KCNQ4* sequences from *O. graminea*, *N. parkeri*, *X. laevis* and *X. tropicalis*. Then, the 5′ and 3′ RACE were performed using a SMARTer RACE cDNA amplification kit and SMARTer RACE kit (TaKaRa, Japan) according to the manufacturer's instructions. The primer information is shown in Tables S1 and S2. The amplified PCR products were purified with a MiniBEST Agarose Gel DNA Extraction Kit (TaKaRa, China), cloned into PMD19‐T vectors, and then sequenced in both directions using an ABI 3730 automated genetic analyzer (Applied Biosystems) by Shanghai Sangon Biological Engineering Technology and Service Co., Ltd. Five to six repeated amplifications were conducted and re‐sequenced to confirm its sequence. The same PCR primers were used for sequencing. All the newly *KCNQ4* sequences were deposited in GenBank under accession numbers: PP179178‐PP179188 (Table 1).

### 2.3 Sequence alignment and statistical analyses

The chromatograms of each sequence were proofread and assembled with the program DNASTAR SeqMan v7.21 (DNASTAR Inc., Madison, WI, USA). All nucleotide sequences from the ORFs of *KCNQ4* and their deduced amino acid sequences were first aligned separately using MUSCLE v3.8 implemented in MEGA v5.0 (Tamura et al., 2011) under default settings, and then manually adjusted with GeneDoc. The nucleotide sequence alignment was generated based on the protein sequence alignment. Two datasets, i.e., the nucleotide and amino acid datasets for the 15 anuran species, were generated and used in the subsequent analyses. A three‐dimensional domain structure of the anuran *KCNQ4* was predicted using SWISS‐MODEL (http://swissmodel.expasy.org).

Phylogenetic trees were reconstructed using Bayesian inference (BI) in MrBayes v3.1.2 (Huelsenbeck & Ronquist, 2001) and Maximum Likelihood (ML) algorithms in MetaPIGA v2.0 (Helaers & Milinkovitch, 2010) for the two datasets mentioned above. Modeltest (Darriba et al., 2020) were used to select the optimal models based on the Akaike Information Criterion (AIC). The best‐fit substitution models that were selected for the nucleotide dataset and the amino acid dataset were HKY + F + I + G4 and JTT + G4, respectively. Maximum likelihood analyses were conducted using MetaPIGAv2.0 (Helaers & Milinkovitch, 2010) with 1000 metaGA replicate searches. The Bayesian analyses were conducted with four Metropolis‐coupled Markov chain Monte Carlo iterations for 20 million generations with default heating values and trees were sampled every 1000 generation. The first 10% of trees were deleted as the “burn‐in” stage and the remaining trees were used to generate the consensus tree and calculate Bayesian posterior probabilities (PP). The stationarity of the likelihood scores of sampled trees was determined using Tracer v1.4 (Rambaut & Drummond, 2007).

### 2.4 Analysis of selective pressure

The CODEML program in PAML 4.7 (Yang, 2007) was used to estimate the rates of synonymous (*d*_S_) and nonsynonymous substitutions (*d*_N_), as well as the *d*_N_/*d*_S_ ratio (*ω*). The nonsynonymous to synonymous rate ratio *ω* indicates changes in selective pressures, with *ω* = 1, *ω* < 1, and *ω* > 1 corresponding to neutral evolution, purifying and positive selection, respectively. A consensus species tree that included all of the species employed in the present study was inferred from Chen et al. (2013), Chen et al. (2017), Feng et al. (2017), and used as guide tree for the subsequent PAML analysis. First, site models were implemented where *ω* could vary among sites to identify the sites under positive selection in anuran species being examined. Three pairs of site models were tested to reveal whether amphibian KCNQ4 was subjected to positive selection: M1a (nearly neutral: *ω*_0_ < 1 and *ω*_1_ = 1) versus M2a (positive selection: *ω*_0_ < 1, *ω*_1_ = 1 and *ω*_2_ > 1), M7 (beta distribution: 0 < *ω*_0_ < 1) and M8 (positive selection; beta distribution: 0 < *ω*_0_ < 1 and *ω*_1_ > 1), and M8a (nearly neutral; beta distribution: 0 < *ω*_0_ < 1 and *ω*_1_ = 1) versus M8 (Yang et al., 2000, 2005). Second, a combination of branch, branch‐site and clade models were used to detect whether or not the focal branch had undergone positive selection. The branch‐specific models allow for variable *ω* ratios among branches but invariable *ω* ratios in sites over the whole phylogeny and can be implemented for the study of changes in selective pressures in specific lineages (Yang & Nielsen, 2002). For branch‐specific models, the ‘free‐ratio’ (M1) model (which assumes an independent *ω* ratio for each branch) versus the ‘one‐ratio’ (M0) model (which assumes the same *ω* for all branches) (Yang, 1998), and the two‐ratio model (which allows *ω* to differ between the background and a focal branch) versus M0 model were conducted (Yang & Nielsen, 2002). The branch‐site models allow the *ω* ratio to vary both among sites and among lineages and thus were efficient to detect positive selection that affects only a few sites along a few lineages (Yang & Nielsen, 2002). Modified branch‐site model A (test 2) (model = 2, Nsites = 2) was performed in each focal lineage (details on test 2 in Zhang et al., 2005). The null model for test 2 is the branch‐site model A with *ω*_2_ = 1 fixed. In addition, the clade model C versus M1a was used to detect divergent selection acting on groups of related key taxa. The clade Model C includes two clades (i.e., focal clade and background clade) and three site classes (0, 1, and 2). Purifying selection (0 < *ω*_0_ < 1) and neutral selection (*ω*_1_ = 1) were assumed in site class 0 and 1, whereas branches in the two clades are evolving with *ω*_2_ and *ω*_3_ (*ω*_2 ≠_ *ω*_3_) in site class 2, respectively. Clade models were separately performed for the ancestral *O. tormota* and *O. graminea*, *Odorrana*, and Neobatrachia. Significance for differences between two nested models were evaluated using likelihood ratio tests (LRTs) by calculating twice the log‐likelihood (2ΔL) difference following a chi‐square distribution, with the number of degrees of freedom equal to the difference in the numbers of free parameters between models. All of the positively selected sites were characterized using a Bayes Empirical Bayes (BEB) analysis with posterior probabilities ≥.80 (Yang, 2007).

Third, the improved statistical methods in Datamonkey web server (Pond & Frost, 2005), which computed nonsynonymous and synonymous substitutions at each codon position, was used to further test the selection. The fixed‐effect likelihood (FEL) and random effect likelihood (REL) models were analyzed. The *ω* values in the FEL and REL analysis were calculated based on the *d*_*S*_ whereas the *d*_*S*_ was fixed in the PAML analysis. Sites with *p* values <.1 for FEL, and Bayes factor >50 for REL were considered as candidates under positive selection. Finally, a complementary protein‐level approach was also used in the TreeSAAP (Woolley et al., 2003) to further evaluate the PAML results.

## 3 RESULTS

### 3.1 Characterization of KCNQ4 gene in anurans

**FIGURE 1:**
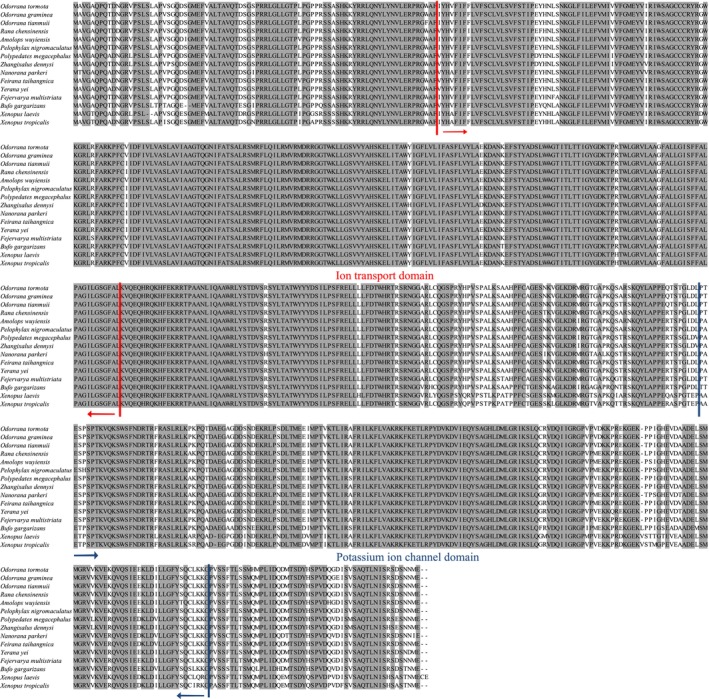
Multiple sequence alignments of the newly obtained 11 anuran KCNQ4 deduced amino acids with other four anurans in GenBank database. The positions of the ion transport domain and potassium ion channel domain are indicated. Similar amino acids are highlighted by gray boxes.

**FIGURE 2:**
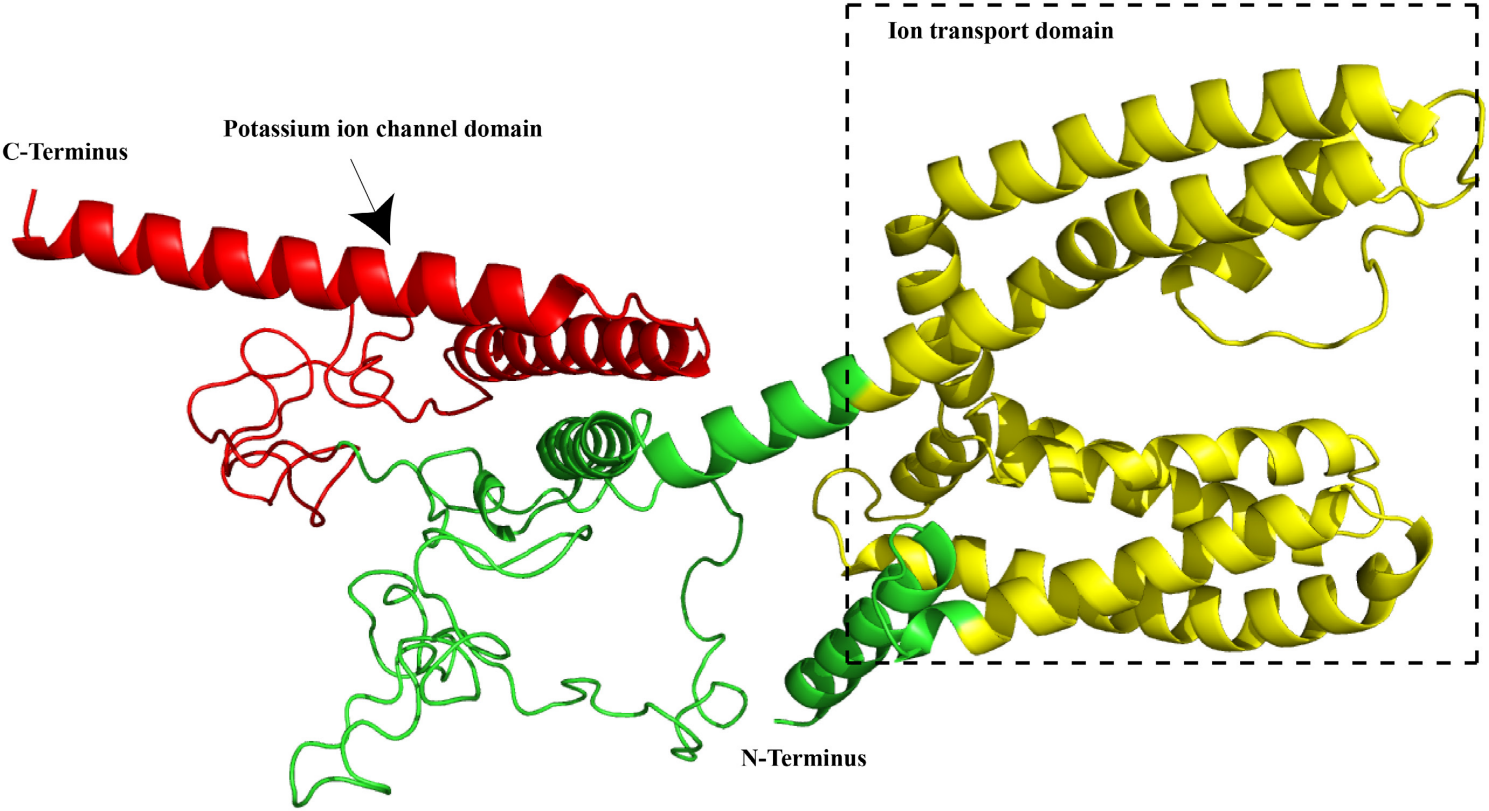
Three‐dimensional structure of the KCNQ4 for *Odorrana tormota*. The ion transport domain and potassium ion channel domains are colored yellow and red, respectively. Amino and carboxy terminal ends are indicated.

In this study, we have successfully amplified and determined the complete ORF sequences for the *KCNQ4* gene in 11 anurans. The full length of the ORF of *KCNQ4* in 10 of the 11 anurans was 2046 bp and it encodes a putative *KCNQ4* protein with 681 amino acids and 76.453 kD molecular weight. In contrast, one single codon deletion was found in *Bufo gargarizans* (Figure 1). No frame‐shift mutations or premature stop codons were found in the ORF of *KCNQ4* in all the 11 anurans. They all shared the typical features of vertebrate *KCNQ4* genes and alignments of the deduced amino acid sequence of the 11 newly obtained and other four anuran *KCNQ4* genes are shown in Figure 1. We found six transmembrane domains (each is composed of 19–25 amino acids) within anuran *KCNQ4* protein as identified in previous studies. The anuran *KCNQ4* protein also possess the characteristic N‐terminal ion transport functional domain (87–311) and the potassium ion channel functional domain (449–631) (Figures 1 and 2), and both domains were highly conserved in all of the anurans examined.

### 3.2 Phylogenetic analyses of KCNQ4 in anurans

The evolutionary history of anuran KCNQ4 was reconstructed using ML and BI methods based on both nucleotide and amino acid datasets. The sampled modern frogs (Neobatrachia) formed a strongly supported monophyletic group (PP = 1.0, BP = 100) from the analyses of both nucleotide and amino acid datasets (Figures S1 and S2). Members of the sampled neobatrachian families (i.e., Ranidae and Rhacophoridae) were found to be monophyletic with strong support from both datasets. The monophyly of Dicroglossidae was strongly supported based on the nucleotide dataset, whereas it was not supported based on the amino acid dataset with the second basal position of *Fejervarya multistriata* among the modern frogs (Figures S1 and S2). Representatives of Dicroglossidae and Ranidae were grouped with weak support and they appeared as the sister taxa to Rhacophoridae. The interrelationships within Ranidae and Dicroglossidae based on the nucleotide dataset were concordant with those in previous studies.

### 3.3 Selective pressure analysis of KCNQ4 in anurans

**FIGURE 3:**
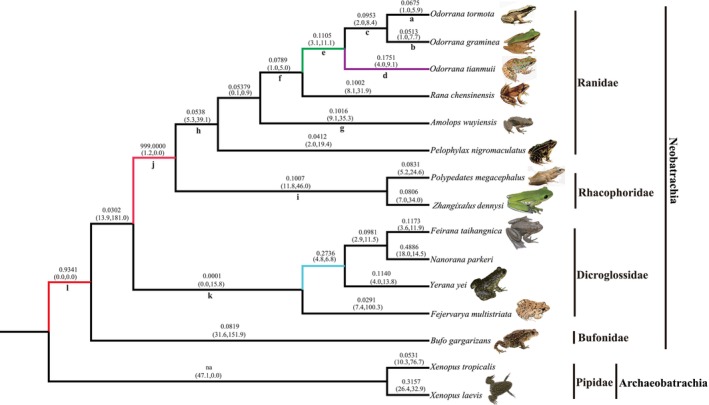
The *ω* values of KCNQ4 genes in distinct evolutionary lineages of anurans. Branches a‐l relate to those shown in Tables S3 and S4. The *ω* values of individual branches shown are based on the free‐ratio model. In some cases, zero synonymous substitutions produced an *ω* value of infinity (n.a.). The estimated numbers of nonsynonymous and synonymous changes are shown in parentheses. The *ω* values of branches e, d, j, and l (marked with different colors) were estimated using the two‐ratio models.

We performed a series of evolutionary models to analyze the selective constraints on anuran KCNQ4, using the species tree shown in Figure 3 as the working topology. The site model analyses showed that models that incorporate selection (i.e., M2a and M8) did not fit the data significantly better than did neutral models (i.e., M1a, M7, and M8a) (Table 2). However, two sites (446L and 478P) were identified to be under selection by the BEB approach as having posterior probabilities ≥.80 with model M8 (Table 2). For the FEL analysis, only one codon (site 574) was identified in the anuran KCNQ4 gene (*d*_N_ */d*_S_ = 12.502, *p* = .0449) (Table 2).

**TABLE 2:** CODEML and FEL analyses of selective pattern on the KCNQ4 gene in anurans.

Models	‐lnL	Models compared	2⊿lnL^a^	d.f.^b^	*p*‐Value^c^	Estimate of parameters	Positively selected sites (*p* ^d^)
Branch models							
M0: one‐ratio	7916.2041					*ω* = 0.0761	Not allowed
M1: free‐ratio	7876.0572	Mo versus M1	80.2938	59	<.001	*ω* variation for each branch	Not allowed
Site models							
M1a (nearly neutral)	7850.756					*p*_0_ = 0.933, *p*_*1*_ = 0.068, *κ* = 2.202, *ω*_0_ = 0.039, *ω*_1_ = 1	Not allowed
M2a (positive selection)	7850.756	M2a versus M1a	0	2	1	*p*_0_ = 0.933, *p*_1_ = 0.047, *p*_2_ = 0.021, *κ* = 2.202, *ω*_0_ = 0.039, *ω*_1_ = 1, *ω*_2_ = 1	66K (.539), 430A (.519), 446L (.591), 478P (.646), 574V (.504), 586P (.519)
M7 (beta)	7841.321					*p* = 0.170, *q* = 1.773, *κ* = 2.153	Not allowed
M8 (beta and omega)	7841.535	M8 versus M7	0.428	2	.807	*p*_0_ = 0.999, *p*_1_ = 0, *p* = 0.164, *q* = 1.663, *κ* = 2.153, *ω* = 1	66K (.746), 87I (.532), 163V (.565), 429S (.538), 430A (.699), 446L (.807), 478P (.885), 574V (.681), 586P (.703)
M8a (beta)	7841.535	M8a versus M8	0	2	1	*p*_0_ = 0.999, *p*_1_ = 0, *p* = 0.164, q = 1.663, *κ* = 2.153, *ω* = 1	
Branch site model							
Ancestral *O. tormota* and *O. graminea* (branch c)							
Alternative	7850.756	Alternative versus Null	0	1	1	*ω*_0_ = 0.039, *ω*_1_ = 1, *ω*_2_ = 1	
Null	7850.756					*ω*_0_ = 0.039, *ω*_1_ = 1, *ω*_2_ = 1	
*Odorrana* (branch e)							
Alternative	7850.738	Alternative versus Null	0	1	1	*ω*_0_ = 0.039, *ω*_1_ = 1, *ω*_2_ = 1	
Null	7850.738					*ω*_0_ = 0.039, *ω*_1_ = 1, *ω*_2_ = 1	
Neobatrachia (branch l)							
Alternative	7850.756	Alternative versus Null	0	1	1	*ω*_0_ = 0.039, *ω*_1_ = 1, *ω*_2_ = 1	
Null	7850.756					*ω*_0_ = 0.039, *ω*_1_ = 1, *ω*_2_ = 1	
Clade model C							
Ancestral *O. tormota* and *O. graminea* (branch c)	7839.110	M1a versus branch c	23.292	3	<.001	*ω*_0_ = 0.026, *p*_0_ = 0.878, *ω*_1_ = 1, *p*_1_ = 0, *ω*_2_ = 0.498, *ω*_3_ = 0.344, *p*_2_ = 0.122	
*Odorrana* (branch e)	7848.455	M1a versus branch e	4.602	3	.203	*ω*_0_ = 0.039, *p*_0_ = 0.931, *ω*_1_ = 1, *p*_1_ = 0.066, *ω*_2_ = 0, *ω*_3_ = 10.654, *p*_2_ = 0.003	
Neobatrachia (branch l)	7837.725	M1a versus branch l	26.062	3	<.001	*ω*_0_ = 0.023, *p*_0_ = 0.864, *ω*_1_ = 1, *p*_1_ = 0, *ω*_2_ = 0.732, *ω*_3_ = 0.428, *p*_2_ = 0.136	
FEL					.0449	*ω* = 12.502	574M

^a^ The log‐likelihood difference between models compared.

^b^ The number of degrees of freedom equal to the difference in the numbers of free parameters between models.

^c^ Likelihood ratio test *p*‐values were adjusted for multiple testing with a Benjamini and Hochberg's procedure and threshold of .05.

^d^ Posterior probabilities of the BEB analysis with *p* > .8 considered as candidates of selection.

In the branch‐specific model analyses, the *ω* ratio calculated in the one‐ratio model (M0) was 0.0761 (Table 2), which indicated the existence of strong functional constraints on anuran KCNQ4 gene. The LRT tests based on the branch model suggested that the free‐ratio model fitted the data better than did the one‐ratio model (*p* < .01, Table 2), indicating the *dN/dS* (*ω*) ratios were different among branches. In addition, no significant evidences of positive selection were detected among all the anuran lineages examined using the two‐ratio models, although the lineage leading to the common ancestor of Ranidae and Rhacophoridae had a *ω* value >1 (Table S3). In contrast, strong significant purifying selection were identified along the lineage leading to the common ancestor of Dicroglossidae (*ω*_1_ = 0.0001, *p* = .0321, Table S3). Interestingly, the lineage leading to the common ancestors of modern frogs (Neobatrachia, branch l in Figure 3) had a higher *ω* value (*ω*_1_ = 0.9341) than the background (*ω*_0_ = 0.0761), although the two ratio models did not fit significantly better than model M0 (Table S3). In addition, the lineages leading to the common ancestors of *O. tianmuii* and the odorous frogs also had a relative higher *ω*_1_ value (0.1755 and 0.1122, respectively) than the background (Table S3).

We then used the branch‐site model to test for positive selection in individual codons for lineages leading to the common ancestor of the two frogs with ultrasonic communication (i.e., *O. tormota* and *O. graminea*) and other groups (Table 2 and Table S4). No significantly positive selection was detected along the lineages leading to the ancestral *O. tormota* and *O. graminea*, *Odorrana* and Neobatrachia (Table 2). The LRT tests showed evidence for positive selection along the lineage leading to the common ancestor of *O. tianmuii* (2ΔL = 7.5816, *p* = .0059), and one codon was detected under positive selection along this lineage with the BEB values of 0.981 (Table S4). In addition, the lineage leading to the common ancestor of Ranidae and Rhacophoridae had a *ω* value of 15.0258, and one positively selected site was identified (BEB value = 0.839), although the LRT test was not significant (Table S4). We subsequently evaluated whether radical changes occurred in the codons under positive selection by using a complementary protein‐level approach implemented in TreeSAAP. The result revealed that both positively selected sites had been through radical changes, and they were involved in or near the N‐terminal ion transport functional domain and the potassium ion channel functional domain (Figures 1 and 2).

Clade models separately undertaken for ancestral *O. tormota* and *O. graminea*, and Neobatrachia revealed evidence of significant divergent selection (Table 2). Of these two clades, the *ω* value of the focal clade was not greater than one, indicating the action of purifying selection on both clades (Table 2). In addition, the *ω* values of the focal clade were lower than those of the background clade for *O. tormota* and *O. graminea* (branch c) (0.344 vs. 0.498), and Neobatrachia (branch l) (0.428 vs. 0.732) (Table 2). However, the *ω* value (10.654) of the focal clade was greater than one for *Odorrana* (branch e) and those of the background clade (Table 2).

## 4 DISCUSSION

Acoustic communication is widespread and plays essential roles in many tetrapods (e.g., frogs, birds and mammals) (Chen & Wiens, 2020). However, the ambient noise has always affected the transmission efficiency of sound information (Vélez et al., 2013). The acquisition of high‐frequency sensitivity among some mammals (e.g., whales, bats and rodents) benefits them via enhancing the signal‐to‐noise ratio to minimal the background noise (reviewed in Vélez et al., 2013). Non‐mammalian vertebrates are restricted in the high‐frequency hearing, with fish, amphibians, and reptiles upper limits of 5 kHz, birds of 8–12 kHz. It is interesting to note that three frog species (i.e., *O. tormota*, *H. cavitympanum* and *O. graminea*) were demonstrated to detect ultrasound (≧20 kHz) and use ultrasonic vocalizations for intraspecific communication (Arch & Narins, 2008; Feng et al., 2006; Shen et al., 2011). The genetic basis that enabled the acquisition of high‐frequency sensitivity in frogs is poorly understood. In this study, we first determined the full‐length cDNA sequences of KCNQ4 in 11 anurans and the evolutionary patterns of KCNQ4 in anurans.

The KCNQ4 gene is highly conserved and has been regarded as strongly functionally constrained during mammalian evolution (Liu et al., 2011, 2012). The alignments of the deduced amino acid sequences of the anuran KCNQ4 genes indicated that the KCNQ4 genes were highly conserved in all of the anurans examined in this study (Figure 1). This strong conservatism of anuran KCNQ4 proteins suggested the important function of KCNQ4 protein in vertebrates (Kharkovets et al., 2006; Lu et al., 2019). The strong functional constraints on anuran KCNQ4 was further confirmed by the analyses of the one‐ratio model (M0) (*ω* = 0.0761). In addition, our results indicated that a generally strong purifying selection of KCNQ4 occurred in most anurans (Figure 3) and suggested that the KCNQ4 gene is functionally important.

Previous studies suggested that the adaptive evolution of KCNQ4 facilitates mammalian hearing and the ultrasonic hearing in bats (Liu et al., 2011, 2012). Analyses of the FEL model revealed one significantly positively site in the anuran KCNQ4 gene (*d*_N_ */d*_S_ = 12.502, *p* = .0449). Evidence of different selective pressures acting on KCNQ4 among different anuran lineages was also detected, which was congruent with the evolutionary patterns of KCNQ4 in bats (Liu et al., 2011, 2012). In addition, the *ω* value (*ω*_1_ = 0.9341) of the lineage leading to the common ancestor of modern frogs (Neobatrachia, branch i in Figure 2) was not significantly higher than 1, but this value was higher than the background (*ω*_0_ = 0.0761) (Table S3). This elevated *ω* estimate (0.9341) for the KCNQ4 gene relative to other branches suggests the accelerated evolution of the KCNQ4 gene in the common ancestor of modern frogs. No significantly positively selection were detected in different anuran lineages based on the branch‐specific and clade model analyses, whereas significantly positively selection were detected along the common ancestor of Ranidae and Rhacophoridae as well as the ancestral *O. tianmuii* (Table 2 and Table S3). *Odorrana tianmuii* inhabits similar habitats with the two ultrasonic frogs and they faced the same ambient noise challenge, which might be an explanation to the positive selection in *O. tianmuii*. Evidence showed that *O. tianmuii* has similar vocal features with *O. tormota*, but the calls of *O. tianmuii* contain no ultrasonic sound. *Odorrana tianmuii* might also use the energy‐saving coping strategies to increase the frequency of calls to resist background noise interference (Shen et al., 2011). In addition, the anuran KCNQ4 might have acquired novel functional capacities and future functional studies should be conducted to confirm this.

However, no significantly positive selection was detected in the common ancestor (branch c in Figure 3) of the two ultrasonic frogs examined in this study (i.e., *O. tormota* and *O. graminea*) based on the branch‐specific, branch‐site model and clade model analyses. Interestingly, analyses of the branch‐site model revealed two positively selected sites, 643H (PP = 0.76) in *O. tormota* (branch a in Figure 3) and 663D (PP = 0.735) in *O. graminea* (branch b in Figure 3). Evidences suggested that only males of *O. tormota* and *O. graminea* have evolved the ultrasonic communication capacity, whereas both females exhibit no ultrasonic sensitivity, which was different from the ultrasonic communication between sexes of bats (Liu et al., 2011, 2012; Shen et al., 2011). This might explain the different evolutionary patterns of KCNQ4 between ultrasonic frogs and bats at some extent. There is accumulating evidence that a series of genes and signal pathway were involved in the ultrasonic communication (Liu et al., 2011, 2012; Pisciottano et al., 2019). Further studies should be focused on other auditory system‐related genes and their relationship to high‐frequency hearing.

## 5 CONCLUSIONS

In the present study, we determined the full‐length cDNA sequences of *KCNQ4* in 11 anurans and explored the structure and evolutionary patterns of *KCNQ4* genes among anurans. Our data suggested that the *KCNQ4* genes were highly conserved in all of the anurans examined in this study and a generally strong purifying selection of *KCNQ4* had occurred in most anurans. Intriguingly, we found evidence of different selective pressures acting on *KCNQ4* among different anuran lineages and significantly positively selection along some anuran lineages. The present study provided valuable information regarding the *KCNQ4* genes in anurans and some new insights for the underpinnings of the high‐frequency hearing in frogs.

## AUTHOR CONTRIBUTIONS

**Yang Guo:** Conceptualization (equal); data curation (equal); formal analysis (lead); methodology (equal); validation (equal); visualization (equal); writing – original draft (lead); writing – review and editing (equal). **Yanjun Zhu:** Data curation (equal); investigation (equal); methodology (equal); resources (equal); validation (equal); writing – original draft (equal). **Shiyuan Shen:** Data curation (equal); investigation (equal); methodology (equal); resources (equal); validation (equal); writing – original draft (equal). **Ningning Lu:** Data curation (equal); investigation (lead); methodology (equal); resources (equal); validation (equal); writing – original draft (equal). **Jie Zhang:** Conceptualization (equal); funding acquisition (equal); investigation (supporting); methodology (equal); project administration (equal); supervision (equal); validation (equal); writing – original draft (equal); writing – review and editing (equal). **Xiaohong Chen:** Conceptualization (equal); data curation (lead); funding acquisition (equal); investigation (equal); methodology (equal); project administration (equal); resources (equal); supervision (equal); validation (equal); writing – original draft (equal); writing – review and editing (lead). **Zhuo Chen:** Conceptualization (lead); data curation (lead); formal analysis (lead); funding acquisition (lead); investigation (lead); methodology (lead); project administration (equal); resources (lead); software (lead); supervision (lead); validation (equal); visualization (equal); writing – original draft (equal); writing – review and editing (lead).

## CONFLICT OF INTEREST STATEMENT

The authors declare that they have no competing interests.

## Supporting information


Figure S1.

Figure S2.

Table S1.

Table S2.

Table S3.

Table S4.

## Data Availability

The data have been deposited in GenBank with the accession number: PP179178–PP179188 and detailed information can be found in Table 1. In addition, the sequences were shown in Figure 1.
