# Supplementary figures and images for: Cloning, characterization, and evolutionary patterns of KCNQ4 genes in anurans
Source: Ecol Evol. 2024 Apr 23;14(4):e11311. doi: 10.1002/ece3.11311 (PMC11036133; doi:10.1002/ece3.11311)

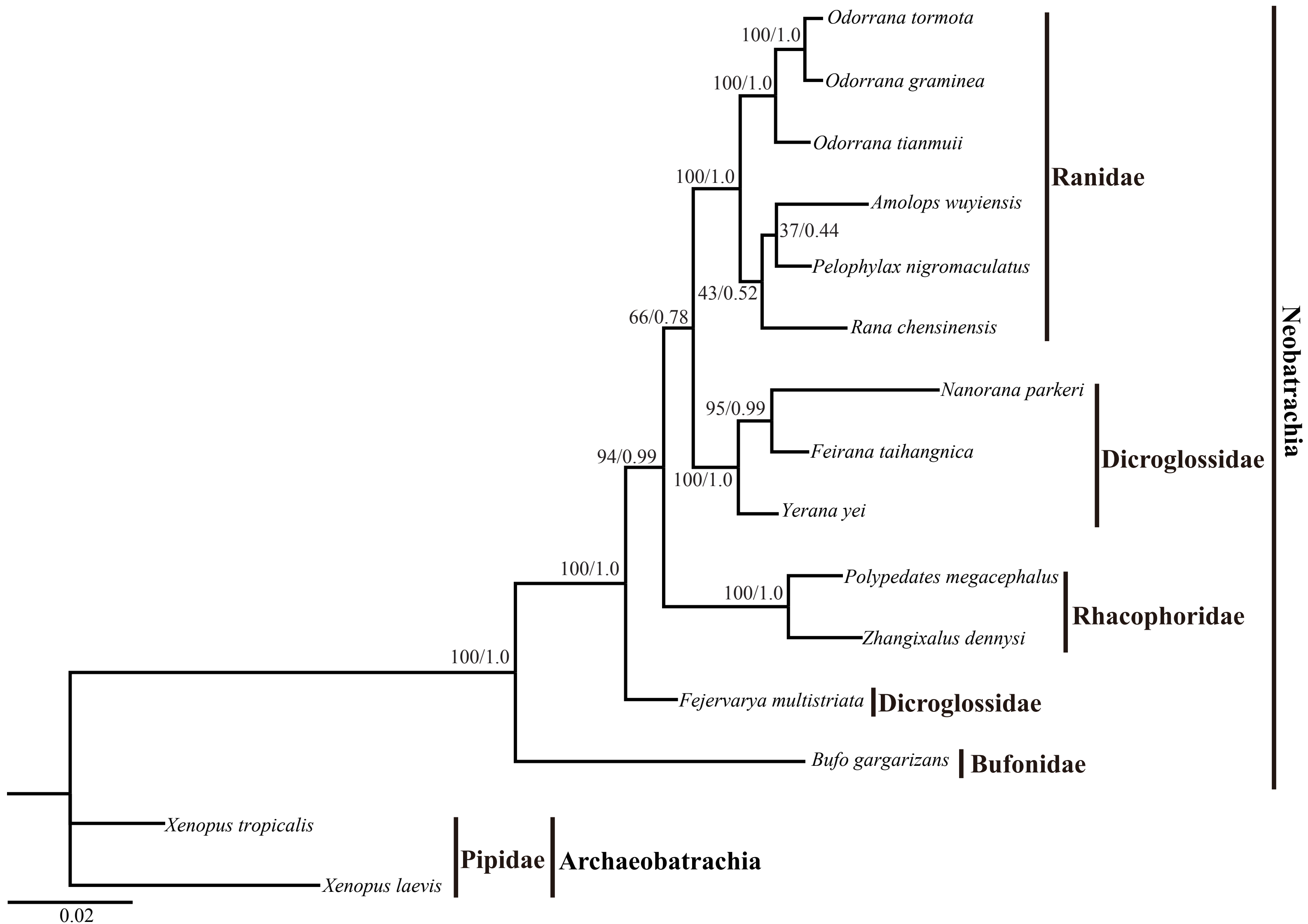

Supplement: Supplementary file 1 — Figure S1. [file ECE3-14-e11311-s003.pdf]

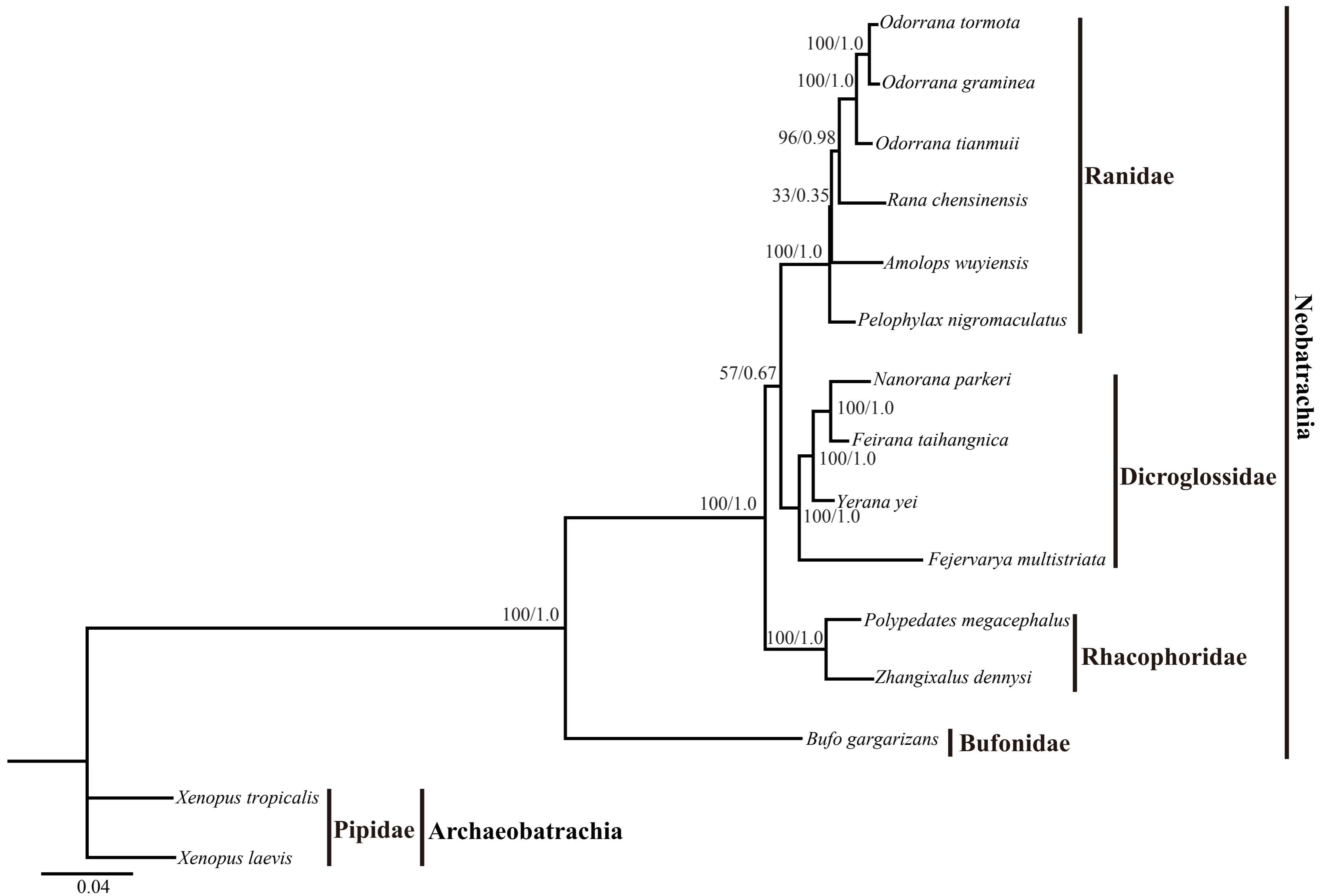

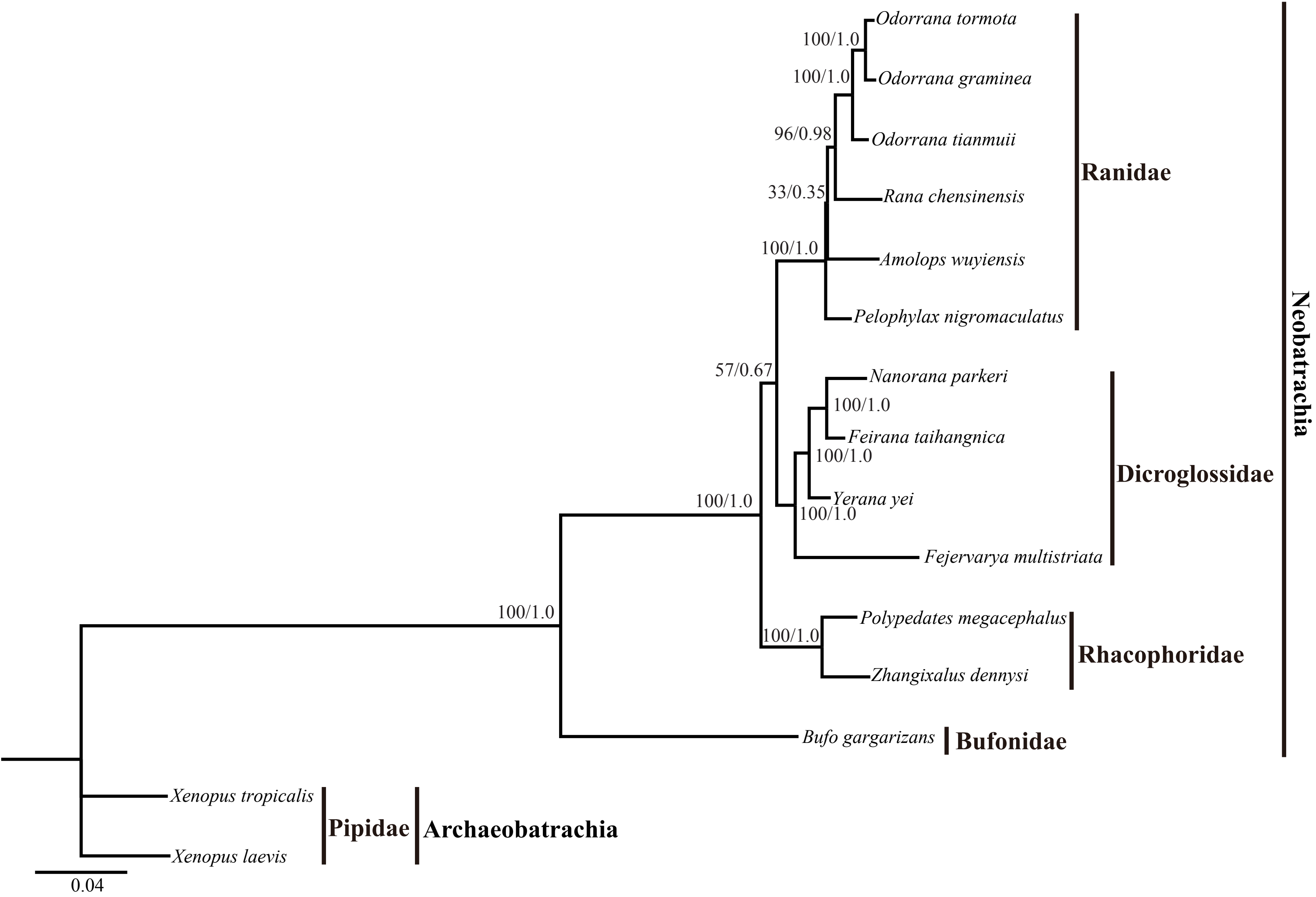

Supplement: Supplementary file 2 — Figure S2. [file ECE3-14-e11311-s002.pdf]
