# Supplementary material for: Cloning, characterization, and evolutionary patterns of KCNQ4 genes in anurans
Source: Ecol Evol. 2024 Apr 23;14(4):e11311. doi: 10.1002/ece3.11311 (PMC11036133; doi:10.1002/ece3.11311)
Supplement: Supplementary file 3 — Table S1. [file ECE3-14-e11311-s004.doc]

Table S1 Primers used for partial cDNA cloning

| **Primer** | **Forward sequence（5’ to 3’）** | **Reverse sequence（5’ to 3’）** | **The species amplified** |
| --- | --- | --- | --- |
| AEK | GTATCCTGCCTTCATTCA | GAGCCTCTTCTCATCATTAC | *Odorrana tormota* |
| TMK | TAGCCTCATTGGCAGTTATT | GGAGCCTCTTCTCATCATTAC | *Odorrana tianmuii* |
| WYK | CTCAGGGTAACATCTTCG | ATCACATCCTTCACATCG | *Amolops wuyiensis* |
| HBK | GGTGCCATCCTTGTCCCT | GAAGCCCGAAACCGTGTA | *Pelophylax nigromaculatus* |
| ZGK | ATGGCAGTGGGGGCACAG | GTCCCAGCTGCAATAACTGCC | *Rana chensinensis* |
| THK | AGGGGCGTCTAAGATTTG | ATTGCTGTCTGAGCGTGA | *Feirana taihangnica*、*Yerana yei* |
| ZLK | AGCATCGCACAAGAGGTA | ACACGAGGAAGGAAGCAA | *Fejervarya multistriata* |
| DSK | CTGGGTTCAGTGGTGTATG | AAGCCCGAAATCGTGTA | *Zhangixalus dennysi* |
| BTK | CTGGGTTCAGTGGTGTATG | AAGCCCGAAATCGTGTA | *Polypedates megacephalus* |
| CCK | CTCCCTGACGCCTACT | CAGAACCCAGAATACCC | *Bufo gargarizans* |

Note: R=A/G，Y=C/T，M=A/C，K=G/T，S=C/G，W=A/T，H=A/C/T，B=C/G/T，V=A/C/G， D=A/G /T，N=A/C/G/T
