# Supplementary material for: Cloning, characterization, and evolutionary patterns of KCNQ4 genes in anurans
Source: Ecol Evol. 2024 Apr 23;14(4):e11311. doi: 10.1002/ece3.11311 (PMC11036133; doi:10.1002/ece3.11311)
Supplement: Supplementary file 4 — Table S2. [file ECE3-14-e11311-s006.docx]

Table S2 Primers used for 3’/5’ RACE

| **Primer** | **Sequence（5’ to 3’）** |
| --- | --- |
| ADK-5’-Out | AGCACCCAGCCGACCAGAT |
| ADK-5’-In | ATGCCAAACACGACAATCA |
| WYK-5’-Out | GCGAAGATGTTACCCTGAGTT |
| WYK-5’-In | AGCACCCAGCCGACCAGAT |
| BTK-5’-Out | GTATGAATAAGCCTTTGTTGGA |
| BTK-5’-In | AGGGACAAGGAGGGCAACC |
| YSK-3’-Out | AGGAAATAATGCCAACTGT |
| YSK-3’-In | TGAGTATGATGGGAAGAGTG |
| 3’RACE Olig(T)-Adaptor^*^ | CTGATCTAGAGGTACCGGATCCTTTTTTTTTTTTTT |
| 3’RACE Adaptor^*^ | CTGATCTAGAGGTACCGGATCC |
| 5’RACE Olig(T)-Adaptor^*^ | GACTCGAGTCGACATCGATTTTTTTTTTTTTTTTT |
| 5’RACE Adaptor^*^ | GACTCGAGTCGACATCG |
