# Supplementary material for: Cloning, characterization, and evolutionary patterns of KCNQ4 genes in anurans
Source: Ecol Evol. 2024 Apr 23;14(4):e11311. doi: 10.1002/ece3.11311 (PMC11036133; doi:10.1002/ece3.11311)
Supplement: Supplementary file 5 — Table S3. [file ECE3-14-e11311-s005.docx]

**Table S3.** Likelihood values and parameter estimates of branch specific models for the *KCNQ4* within Anurans.

| **Models** | **ln L^a^** | **Estimate of parameters** | **2ΔL^b^** | ***P*-value** |
| --- | --- | --- | --- | --- |
| **Branch-specific models** |  |  |  |  |
| **Branch a (*O. tormota*)** |  |  |  |  |
| Two-ratio | -7916.1960 | ω_0_ = 0.0762, ω_1_ = 0.0664 | 0.0162 | 0.8987 |
| **Branch b (*O. graminea*)** |  |  |  |  |
| Two-ratio | -7916.1318 | ω_0_ = 0.0763, ω_1_ = 0.0516 | 0.1446 | 0.7038 |
| **Branch c (ancestral *O. tormota* and *O.graminea*)** |  |  |  |  |
| Two-ratio | -7916.1647 | ω_0_ = 0.0760, ω_1_ = 0.0957 | 0.0788 | 0.7789 |
| **Branch d (ancestral *O. tianmuii*)** |  |  |  |  |
| Two-ratio | -7915.3645 | ω_0_ = 0.0753, ω_1_ = **0.1755** | 1.6792 | 0.1950 |
| **Branch e (ancestral *Odorrana*)** |  |  |  |  |
| Two-ratio | -7916.0468 | ω_0_ = 0.0758, ω_1_ = **0.1122** | 0.3146 | 0.5749 |
| **Branch f (ancestral *Odorrana* and *Rana*)** |  |  |  |  |
| Two-ratio | -7916.2015 | ω_0_ = 0.0761, ω_1_ = 0.0826 | 0.0052 | 0.9425 |
| **Branch g (ancestral *A. wuyiensis*)** |  |  |  |  |
| Two-ratio | -7915.9126 | ω_0_ = 0.0753, ω_1_ = 0.1019 | 0.5830 | 0.4451 |
| **Branch h (ancestral Ranidae)** |  |  |  |  |
| Two-ratio | -7916.0370 | ω_0_ = 0.0767, ω_1_ = 0.0588 | 0.3342 | 0.5632 |
| **Branch i (ancestral Rhacophoridae)** |  |  |  |  |
| Two-ratio | -7915.9099 | ω_0_ = 0.0751, ω_1_ = 0.0994 | 0.5884 | 0.4430 |
| **Branch j (ancestral Ranidae and Rhacophoridae)** |  |  |  |  |
| Two-ratio | -7915.5964 | ω_0_ = 0.0757, ω_1_ =**999.0000** | 1.2154 | 0.2703 |
| **Branch k (ancestral Dicroglossidae)** |  |  |  |  |
| Two-ratio | -7913.9087 | ω_0_ = 0.0771, ω_1_ = **0.0001** | 4.5908 | **0.0321** |
| **Branch l (ancestral Neobatrachia)** |  |  |  |  |
| Two-ratio | -7916.2045 | ω_0_ = 0.0761, ω_1_ = **0.9341** | 0.0008 | 0.9774 |

^a^ln L is the log-likelihood score.

^b^likelihood ratio test (LRT) to detect positive selection.

The ω values larger than 1 and those relative higher than the background are shown in boldface. The significant P-value is shown in boldface.
