# Supplementary material for: Cloning, characterization, and evolutionary patterns of KCNQ4 genes in anurans
Source: Ecol Evol. 2024 Apr 23;14(4):e11311. doi: 10.1002/ece3.11311 (PMC11036133; doi:10.1002/ece3.11311)
Supplement: Supplementary file 6 — Table S4. [file ECE3-14-e11311-s001.docx]

**Table S4.** Likelihood values and parameter estimates of branch site model for the *KCNQ4* gene within Anurans.

| **Models** | **ln L^a^** | **Estimate of parameters** | **2ΔL^b^** | ***P*-value** | **Positively selected sites (P^c^)** |
| --- | --- | --- | --- | --- | --- |
| **Branch a (*O. tormota*)** |  |  |  |  |  |
| Alternative | -7850.5754 | ω_0_ = 0.0387, ω_1_ = 1, ω_2_ = 1 | 0 | 1.0 | 643H(0.76) |
| Null | -7850.5754 | ω_0_ = 0.0387, ω_1_ = 1, ω_2_ = 1 |  |  |  |
| **Branch b (*O. graminea*)** |  |  |  |  |  |
| Alternative | -7850.6589 | ω_0_ = 0.0387, ω_1_ = 1, ω_2_ = 1 | 0 | 1.0 | 663D(0.735) |
| Null | -7850.6589 | ω_0_ = 0.0387, ω_1_ = 1, ω_2_ = 1 |  |  |  |
| **Branch c (ancestral *O. tormota* and *O.graminea*)** |  |  |  |  |  |
| Alternative | -7850.7555 | ω_0_ = 0.0390, ω_1_ = 1, ω_2_ = 1 | 0 | 1.0 |  |
| Null | -7850.7555 | ω_0_ = 0.0390, ω_1_ = 1, ω_2_ = 1 |  |  |  |
| **Branch d (ancestral *O. tianmuii*)** |  |  |  |  |  |
| Alternative | -7845.6765 | ω_0_ = 0.0387, ω_1_ = 1 , ω_2_ = **999.0000** | 7.5816 | **0.0059** | **84W(0.981)** |
| Null | -7849.4673 | ω_0_ = 0.0387, ω_1_ = 1, ω_2_ = 1 |  |  |  |
| **Branch e (ancestral *Odorrana*)** |  |  |  |  |  |
| Alternative | -7850.7384 | ω_0_ = 0.0389, ω_1_ = 1, ω_2_ = 1 | 0 | 1.0 | 575D(0.697) |
| Null | -7850.7384 | ω_0_ = 0.0389, ω_1_ = 1, ω_2_ = 1 |  |  |  |
| **Branch f (ancestral *Odorrana* and *Rana*)** |  |  |  |  |  |
| Alternative | -7850.7555 | ω_0_ = 0.0390, ω_1_ = 1, ω_2_ = 1 | 0 | 1.0 |  |
| Null | -7850.7555 | ω_0_ = 0.0390, ω_1_ = 1, ω_2_ = 1 |  |  |  |
| **Branch g (ancestral *A. wuyiensis*)** |  |  |  |  |  |
| Alternative | -7850.7555 | ω_0_ = 0.0390, ω_1_ = 1, ω_2_ = 1 | 0.0796 | 0.7778 | 27S(0.589); 432S(0.583); 626S(0.604) |
| Null | -7850.7157 | ω_0_ = 0.0388, ω_1_ = 1, ω_2_ = 1 |  |  |  |
| **Branch h (ancestral Ranidae)** |  |  |  |  |  |
| Alternative | -7850.7555 | ω_0_ = 0.0390, ω_1_ = 1, ω_2_ = 1 | 0 | 1.0 |  |
| Null | -7850.7555 | ω_0_ = 0.0390, ω_1_ = 1, ω_2_ = 1 |  |  |  |
| **Branch i (ancestral Rhacophoridae)** |  |  |  |  |  |
| Alternative | -7850.3694 | ω_0_ = 0.0383, ω_1_ = 1, ω_2_ = 1 | 0 | 1.0 | 110E(0.559); 128V(0.571); 605V(0.583); 666V(0.553) |
| Null | -7850.3694 | ω_0_ = 0.0383, ω_1_ = 1, ω_2_ = 1 |  |  |  |
| **Branch j (ancestral Ranidae and Rhacophoridae)** |  |  |  |  |  |
| Alternative | -7849.5788 | ω_0_ = 0.0385, ω_1_ = 1, ω_2_ = **15.0258** | 0.0634 | 0.8012 | **482T(0.839)** |
| Null | -7849.6105 | ω_0_ = 0.0385, ω_1_ = 1, ω_2_ = 1 |  |  |  |
| **Branch k (ancestral Dicroglossidae)** |  |  |  |  |  |
| Alternative | -7850.7555 | ω_0_ = 0.0390, ω_1_ = 1, ω_2_ = 1 | 0 | 1.0 |  |
| Null | -7850.7555 | ω_0_ = 0.0390, ω_1_ = 1, ω_2_ = 1 |  |  |  |
| **Branch l (ancestral Neobatrachia)** |  |  |  |  |  |
| Alternative | -7850.7555 | ω_0_ = 0.0390, ω_1_ = 1, ω_2_ = 1 | 0 | 1.0 |  |
| Null | -7850.7555 | ω_0_ = 0.0390, ω_1_ = 1, ω_2_ = 1 |  |  |  |

^a^ ln L is the log-likelihood score.

^b^ likelihood ratio test (LRT) to detect positive selection.

^c^ Posterior probabilities of the BEB analysis with p >0.8 considered as candidates of selection and those with p>0.8 are shown in boldface.

The ω values larger than 1 are shown in boldface.
